# Automatic Multi-Camera Extrinsic Parameter Calibration Based on Pedestrian Torsors †

**DOI:** 10.3390/s19224989

**Published:** 2019-11-15

**Authors:** Anh Minh Truong, Wilfried Philips, Nikos Deligiannis, Lusine Abrahamyan, Junzhi Guan

**Affiliations:** 1 TELIN-IPI, Ghent University—imec, St-Pietersnieuwstraat 41, B-9000 Gent, Belgium; Wilfried.Philips@UGent.be; 2 ETRO Department, Vrije Universiteit Brussel—imec, Pleinlaan 2, B-1050 Brussels, Belgium; ndeligia@etrovub.be (N.D.); alusine@etrovub.be (L.A.); 3 CETC Key Laboratory of Aerospace Information Applications, Shijiazhuang 050000, China; guanjunzhi@hotmail.com

**Keywords:** extrinsic calibration, camera network, pedestrians

## Abstract

Extrinsic camera calibration is essential for any computer vision task in a camera network. Typically, researchers place a calibration object in the scene to calibrate all the cameras in a camera network. However, when installing cameras in the field, this approach can be costly and impractical, especially when recalibration is needed. This paper proposes a novel, accurate and fully automatic extrinsic calibration framework for camera networks with partially overlapping views. The proposed method considers the pedestrians in the observed scene as the calibration objects and analyzes the pedestrian tracks to obtain extrinsic parameters. Compared to the state of the art, the new method is fully automatic and robust in various environments. Our method detect human poses in the camera images and then models walking persons as vertical sticks. We apply a brute-force method to determines the correspondence between persons in multiple camera images. This information along with 3D estimated locations of the top and the bottom of the pedestrians are then used to compute the extrinsic calibration matrices. We also propose a novel method to calibrate the camera network by only using the top and centerline of the person when the bottom of the person is not available in heavily occluded scenes. We verified the robustness of the method in different camera setups and for both single and multiple walking people. The results show that the triangulation error of a few centimeters can be obtained. Typically, it requires less than one minute of observing the walking people to reach this accuracy in controlled environments. It also just takes a few minutes to collect enough data for the calibration in uncontrolled environments. Our proposed method can perform well in various situations such as multi-person, occlusions, or even at real intersections on the street.

## 1. Introduction

Extrinsic camera calibration provides the coordinate system transformations from 3D world coordinates to 3D camera coordinates for all the cameras in the network. This information is essential for many machine vision applications such as tracking, augmented reality, free view image synthesis, 3D reconstruction [1,2,3], or transferring the well-trained recognition models to different camera setups [4]. The classical methods [5,6,7] require a sufficient number of point correspondences of calibration objects to estimate the extrinsic parameters accurately. In addition, the calibration objects also have to be well-observed among all cameras. Moreover, calibrating cameras without any mistakes by using classical methods requires a certain level of skill while sending skilled technicians onsite to recalibrate cameras is costly and time-consuming. It also would be even worse because the cameras also need to be recalibrated after the cameras are adjusted or moved. Additionally, this traditional approach does not work for historic multi-camera video sequences in which no calibration objects were recorded.

Hartley et al. [8] proposed an auto-calibration method based on scene reconstruction from arbitrary features. Due to the interactive fashion as well as a large number of parameters has to be estimated, this method is slow and not always be able to achieve reliable results. Analyzing human data in images and video is the main concern of many machine vision applications. Thus, to leverage the information which also is extracted to serve other high-level tasks, many autocalibration methods [9,10,11,12,13,14] based on pedestrians are proposed. However, they are sensitive to noise as well as impractical for several situations in practice.

The proposed method relies on finding humans in images and estimating their centerline. For this purpose, we use OpenPose [15,16]. Human pose estimation is also an important task for various machine vision applications, such as action recognition, motion capture, sports, etc. Many real-time human estimation methods [15,16,17,18,19,20,21] have been proposed in recent years. Therefore, human pose estimation is fast enough for use in extrinsic calibration. Moreover, pose estimation is useful in itself for video analytics. Therefore, if an application already includes pose estimation, we can reuse this “for free” in calibration. Note that, in this paper, we assume the intrinsic camera parameters have been estimated before the extrinsic camera calibration.

The first contribution of our paper is that we replace the ellipse based detection of the top and the bottom of pedestrians by an approach based on modern human pose estimators. Thus, it provides more robust detection of people and a more accurate estimation of their centerlines. Then, we analyze the amount of video of walking people it needs to reach the desired accuracy. We also combine the proposed method calibration method with a random sampling strategy to deal with the noise and outliers in the estimated human pose data. It shortens the required time of data collection, and also makes our method robust to outliers and noise.

The second contribution is that we propose an automatic method to also handle the case of multiple pedestrians simultaneously in the scene. In [14], this case was handled by manual annotation. In the paper, we propose a brute-force, but still fast, method to effectively find the correspondences and also eliminate correspondences that have poor estimated human pose. We show that it produces accurate results and more complicated methods are not needed.

The third contribution, we proposed a novel extrinsic calibration method based on just the information of the top and the centerlines of the pedestrian. Thus, it helps the proposed calibration system can work well even without the information of the bottom of the pedestrians (which usually happens in heavily occluded scenes). Therefore, the proposed method can be applied to a wide range of usage scenarios, including indoor and outdoor scenes. The experimental results show that the proposed method can achieve very precise accuracy in many challenging scenarios including real intersection, heavily occluded, and multiple people scenes.

The rest of the paper is organized as follows. We discuss the related work in Section 1. In Section 2, we describe the architecture of our calibration method for a pair of cameras in detail. In Section 3, we explain the way to extend the proposed method for a camera network as well as the novel extrinsic calibration based on just the top and centerline of the walking people. We present the obtained results and the detailed analysis of our experiments in Section 4. Finally, we discuss the conclusion and future work in Section 5.

## 2. Related Work

Lv et al. [12,22] detect and select the walking human from video sequences by the transition of foreground object shapes. They represent the pedestrians as vertical “walking sticks” of the same height in the 3D environment. Then, they compute the vertical vanishing point and the horizon line based on the vertical “walking sticks”. Li et al. [11] proposed a single view camera calibration method that directly estimates the focal length, the tilting angle, and the camera height by using a nonlinear regression model from the observed head and feet points of a walking human.

In [9], Liu et al. proposed a fully automatic calibration method for monocular stationary cameras. They leverage relative 3D pedestrian height distribution to eliminate false pedestrian detections in moderately crowded scenes. In [10], Liu et al. extended their earlier work to camera network calibration. Iteratively, they incorporate robust matching with a partial direct linear transform. Due to the reliance on vanishing point (intersection of near-parallel lines) estimation, a small error of head (or feet) detection could lead to a big error of extrinsic parameters. On the other hand, our method estimates the extrinsic parameters based on estimated 3D positions of the head and feet which is much less sensitive to noise. Moreover, their methods cannot work if there is no feet information in the heavily occluded scene while the proposed method still performs well in that situation. In [23], Lucas et al. proposed a method for urban areas by combining the information from the pedestrians and structures that have parallel and orthogonal lines, such as buildings and road lines. Method [23] addresses the information of pedestrians in a similar manner as [10], and it is only applicable to single view applications.

Most methods [9,10,12] assume that moving pedestrians walk on a planar, horizontal surface. Possegger et al. [13] proposed an unsupervised extrinsic self-calibration method for a network of static cameras and pan-tilt-zoom cameras solely based on correspondences between tracks of a walking human. Then, they eliminate the outliers of feet and head detection by estimating pairwise homographies between the camera views based on the detected locations of feet and head. Finally, they compute the extrinsic parameters of the cameras by solving a non-linear optimization problem to minimize the reprojection error. Therefore, it tends to get stuck in local optima without a good initialization which was not presented in their work. In contrast, our method can have a precise estimated 3D position of the head and feet based on a robust human pose detector for the extrinsic calibration. Our method also does not require the person to walk on a plane surface.

Hödlmoser et al. [24] proposed a novel method to estimate the essential matrix based on the locations of the feet and head of the single pedestrian from the video sequences. After that, the extrinsic parameters were extracted by essential matrix decomposition. To find out the unique and proper solution for the rotation parameters and translation parameters, they needed to apply the chirality check [25,26]. Therefore, their method is quite sensitive to erroneous of the estimated locations between head (or foot) correspondences in different camera views. If the pedestrian walks along a straight line which occurs quite often in practice, all head and foot positions lie in the same plane. This leads to the degenerate case for the essential matrix estimation [27]. Therefore, they can only obtain the homography matrix between two views. However, in this case, their method cannot obtain the unique extrinsic parameters by decomposing the homography matrix [28,29]. Thus, it cannot find the extrinsic parameters with reasonable accuracy in this case.

Our paper is based on the work of Guan et al. [14]; our method does not require that the pedestrian walks on a plane surface (e.g., walking on steps and stairs), as long as the posture of the pedestrian remains the same while walking. The method obtains the extrinsic parameters by computing the 3D rigid body transformation that optimally aligns two sets of points corresponding to top and bottom of the pedestrian. The correspondence of these points between camera views is assumed to be known. In practice, this method, therefore, requires manual annotations to differentiate between multiple people and is not fully automatic. In [14], the top and bottom detection was implemented based on change detection and is not very robust w.r.t. noise and occlusion. In contrast, our propose a method is fully automated and uses a more robust human pose detector. In [30], Lettry et al. proposed a method to solve correspondences for camera calibration based on multiple pedestrians. However, their method produces incorrect correspondences which degrade the accuracy of the calibration.

## 3. Proposed Method

**Figure 1:**
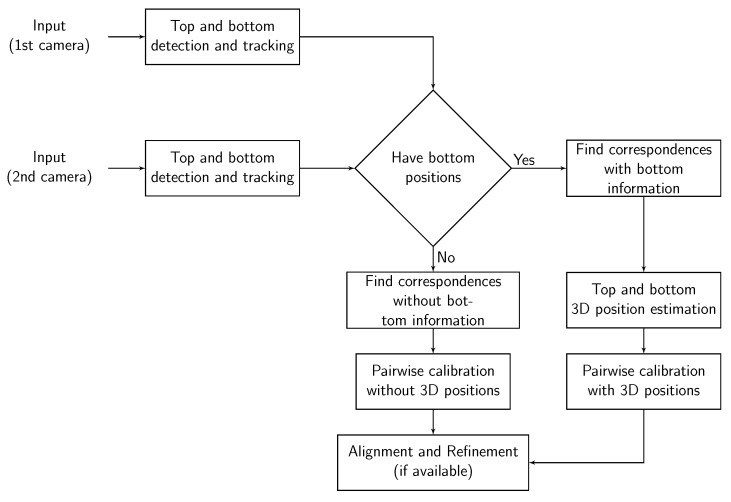
Block scheme of the proposed calibration method.

Figure 1 shows the block diagram of the proposed framework of multi-camera calibration based on walking pedestrians. First, the proposed method calibrates all the cameras in the network in a pairwise fashion. Then, if the ground truth measurements are available (at least 3 points), we can apply the refinement and alignment to further optimize the extrinsic parameters. If the bottom of the pedestrians cannot be observed, we apply the novel method to estimate the extrinsic parameters based on just the top and the centerline of the walking person (Section 3.5). However, if the bottom of the pedestrians can be observed properly, we apply the extrinsic calibration method based on the top and the bottom that extracted from OpenPose [16] to have more accurate extrinsic parameters.

### 3.1. Extract the Positions of the Top and the Bottom of the the Observed Pedestrian in Image Coordinates

First, we assume that the frame synchronization, as well as the intrinsic calibration for all cameras in the network, have been done before the extrinsic calibration. To obtain the position of a walking person in the image, we apply the human pose estimation method in [16]. This produces a skeleton model of all major body joints. Because the locations of the head joints are not stable enough in these skeletons, we use the neck joint locations instead as the top of the observed pedestrian.

In this work, we compute the bottom of the observed pedestrian in two different ways. The first one is the midpoint of the left ankle joint and the right ankle joint as the bottom position of a walking person. However, the ankle of the walking person does not always appear in the mildly occluded scene. However, the hip’s joints of the walking person could be well observed in the mildly occluded scene. Thus, we also investigate to extract the bottom part of the body by the middle point of the left hip joint and the right hip joint.

In the case that the bottom of the pedestrians cannot be observed, to determine the image positions of the top and the centerline of a walking person, we propose to detect the bounding boxes of the walking person (are extracted by YOLO [31]) in the first step. We estimate the centerline of a pedestrian by the line from the center of the top edge to the center bottom edge of the bounding box.

### 3.2. Extrinsic Camera Calibration Based on a Pedestrian

Consider a camera network with *K* cameras $C_{1},C_{2},...,C_{K}$. Let $r^{\left(w\right)}={\left(X_{w},Y_{w},Z_{w}\right)}^{T}$ be a point in a 3D world coordinate system that is visible to the camera, where the superscript *T* denotes a matrix transposition. In our work, each camera in the camera network has its own distinct camera coordinate system. We denote a 3D point in the coordinate system of camera *k* as $r^{\left(k\right)}={\left(X^{\left(k\right)},Y^{\left(k\right)},Z^{\left(k\right)}\right)}^{T}$. Without loss of generality, we choose the coordinate system of the camera $C_{1}$ as the world coordinate system as follows:

(1) $$r^{\left(w\right)}=r^{\left(1\right)}.$$

Thus, we can present the transformation between the camera coordinates $r^{\left(k\right)}$ and the world coordinates $r^{\left(w\right)}$ as follows:

(2) $$r^{\left(k\right)}=R^{\left(k\right)}r^{\left(w\right)}+c^{\left(k\right)},$$

where $c^{\left(k\right)}$ are the coordinates of the origin of the global world coordinate system with regards to the local coordinate system of camera *k*. $R^{\left(k\right)}$ is the rotation matrix which is a 3×3 matrix.

To obtain the extrinsic parameters for all cameras in the camera network, we first calibrate the camera network in a pairwise fashion. Thus, let us consider a camera system (which is composed of two cameras) where a person moving between *N* different locations while keeping a fixed posture (the bottom and the top of the person can be observed from both cameras). Let ${\tilde{u}}_{\mathrm{bottom}}^{\left(k\right)}\left(t\right)$ and ${\tilde{u}}_{\mathrm{top}}^{\left(k\right)}\left(t\right)$ be the image positions of the bottom (feet or hip) and top (neck) at the *t*-th locations in camera *k* (where k∈{1,2}). Let ${\tilde{x}}_{\mathrm{bottom}}^{\left(k\right)}\left(t\right)$, and ${\tilde{x}}_{\mathrm{top}}^{\left(k\right)}\left(t\right)$ be the normalized image coordinates (x,y,1) of the bottom and the top, respectively. We obtain the unknown *Z* coordinates of the bottom $Z_{\mathrm{bottom}}^{\left(k\right)}\left(t\right)$ and *Z* coordinates of the top $Z_{\mathrm{top}}^{\left(k\right)}\left(t\right)$ for camera *k* by applying the proposed method in [14]. Suppose that person walks upright and has heigh *h* where *h* is measured from the top of the pedestrian to the bottom of the pedestrian. Let $r_{\mathrm{top}}^{\left(k\right)}\left(t\right)=Z_{\mathrm{top}}^{\left(k\right)}\left(t\right){\tilde{x}}_{\mathrm{top}}^{\left(k\right)}\left(t\right)$ and $r_{\mathrm{bottom}}^{\left(k\right)}\left(t\right)=Z_{\mathrm{bottom}}^{\left(k\right)}\left(t\right){\tilde{x}}_{\mathrm{bottom}}^{\left(k\right)}\left(t\right)$ be the 3D camera coordinates of the top and bottom. Thus, we have:

(3) $$r_{\mathrm{top}}^{\left(k\right)}\left(t\right)-r_{\mathrm{bottom}}^{k}\left(t\right)=Z_{\mathrm{top}}^{\left(k\right)}\left(t\right){\tilde{x}}_{\mathrm{top}}^{\left(k\right)}\left(t\right)-Z_{\mathrm{bottom}}^{\left(k\right)}\left(t\right){\tilde{x}}_{\mathrm{bottom}}^{\left(k\right)}\left(t\right)=he_{z}^{\left(k\right)},$$

where $e_{z}^{\left(k\right)}$ is unit vector of the person within camera *k*.

From ${\tilde{x}}_{\mathrm{bottom}}^{\left(k\right)}\left(t\right)$ and ${\tilde{x}}_{\mathrm{top}}^{\left(k\right)}\left(t\right)$, it is possible to compute a 3D vector $m^{\left(k\right)}\left(t\right)={\tilde{x}}_{\mathrm{bottom}}^{\left(k\right)}\left(t\right)\times {\tilde{x}}_{\mathrm{top}}^{\left(k\right)}\left(t\right)$ which is perpendicular to the unique vertical plane containing the origin of camera *k*, ${\tilde{x}}_{\mathrm{bottom}}^{\left(k\right)}\left(t\right)$, and ${\tilde{x}}_{\mathrm{top}}^{\left(k\right)}\left(t\right)$. At a given time instant, the intersection of all of those planes is a line along the vertical direction. We could also cancel *h* in ${\left(m^{\left(k\right)}\left(t\right)\right)}^{T}he_{z}^{\left(k\right)}=0$ which leads to Equation (4) because $he_{z}^{\left(k\right)}$ is on the aforementioned plane.

(4) $${\left(m^{\left(k\right)}\left(t\right)\right)}^{T}e_{z}^{\left(k\right)}=0.$$

Therefore, $e_{z}^{\left(k\right)}$ is determined by SVD of matrix $M^{\left(k\right)}={\left(m^{\left(k\right)}\left(t\right)\right)}^{T}$. As explained in [14], once $e_{z}^{\left(k\right)}$ is determined, we can compute the 3D locations of the of the bottom and top w.r.t. by the least-squares solutions up to a constant factor *h*. Finally, we apply the orthogonal Procrustes analysis [32] to estimate the rigid body transformation (relative camera pose in 3D space) between two sets of 3D points (top and bottom).

### 3.3. Robust Extrinsic Calibration

**Figure 2:**
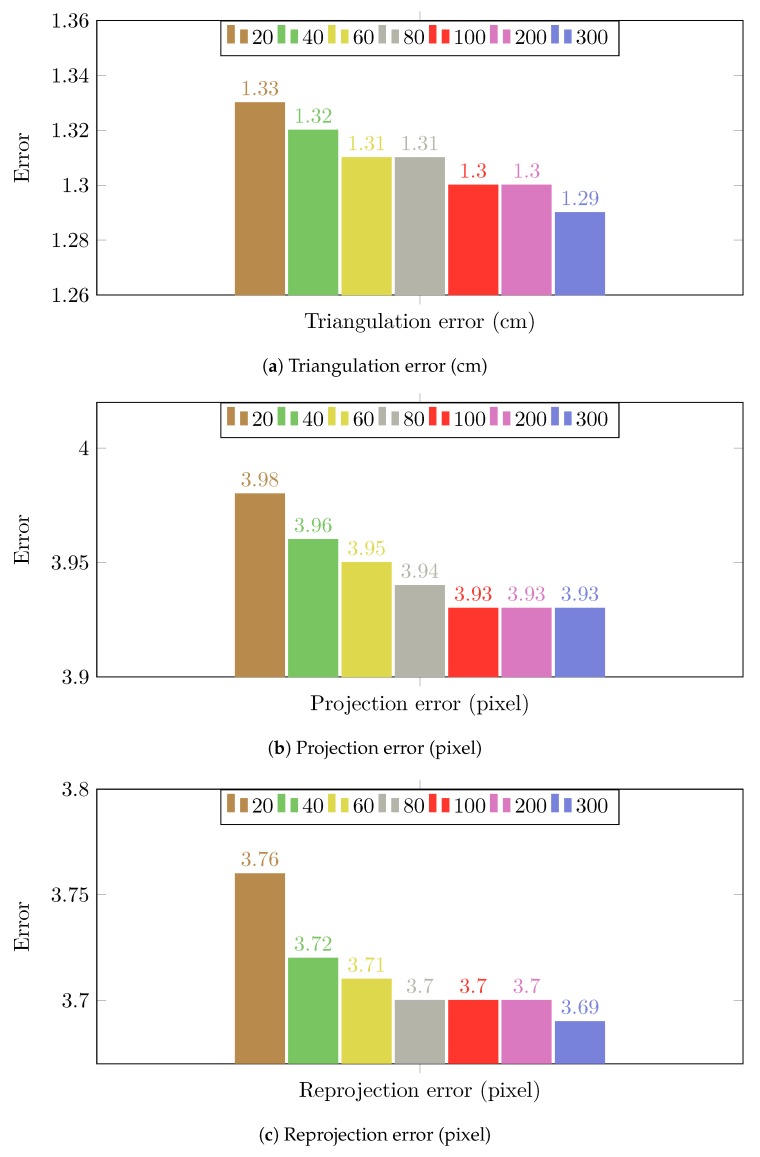
The calibration results of the proposed method on Camera Network 1 (multiple people walking in an empty room) by using different number of random locations. Each location yields two calibration samples (top and bottom).

The proposed calibration method can obtain the appropriate extrinsic parameters when the number of inliers is large enough to compensate for the bad effect of the outliers. However, in practice, we do not always have enough samples to have a precise calibration, e.g., calibrate camera network from videos that were recorded a long time ago. The accuracy of the calibration does not only depend on the number of the samples but also the distribution of locations. For example, using more random locations from different spots of the room helps the proposed method improve accuracy (Figure 2). However, the result of calibration from 20 s (approximately 300 locations) in Table 1 is worse than the result of the calibration from 20 random locations of the room (which is selected randomly from different spots of the scene) in Table 2. It is easy to understand because the person just slowly walks in the room, most of the locations collected in 20 s are so close to each other, and different cameras may see different points of the top or the bottom of the pedestrians. This means many of those locations provide similar information which is not so useful to improve the result of the calibration. Moreover, the outliers of the detection make the information around some spots of the room become inconsistence.

In [14], Guan et al. proposed a method based on RANSAC [33] to obtain a subset of top (or bottom) locations which are likely to be observed similarly in different views. However, the estimated 3D points which satisfy this criterion, do not always agree with the same unit vector $e_{z}^{\left(k\right)}$. Furthermore, the pose of the walking people can slightly change during the video sequences which also makes the unit vector of the centerlines slightly changed. So, including all locations that are likely to be observed similarly cannot effectively improve the calibration results. It can also produce bad $e_{z}^{\left(k\right)}$ estimation which leads to a bad extrinsic calibration. Instead, we need to find an optimal sparse subset of collected locations without the outliers to improve the performance of the proposed method on short video sequences (short amount of time to collect data). Therefore, we propose a random sample scheme as Algorithm 1 to obtain the sparse subset of collected locations which produces the most stable extrinsic calibration (low reprojection error).

**Algorithm 1:** The steps of random sample scheme to calibrate the extrinsic parameter of camera *a* and camera *b*. **Input:**
$H^{\left(a\right)}$ and $H^{\left(b\right)}$—lists of pair locations of top and bottom of the pedestrian in the video sequence, $L^{\left(ab\right)}$—the list of frame indices of key locations (as in Algorithm 2), and the number of repetition *M*.
 **Output:** the extrinsic parameters. **Step 1.** For each frame index *i* in $L^{\left(ab\right)}$, we randomly select a location at frame *t* that is neighbor of it ($t-i<\epsilon_{time}$). In our experiment, we chose the $\epsilon_{time}=10$. Then, we compute extrinsic parameters using the method that we presented in Section 3.2.  **Step 2.** Count the number of pairs agreeing with the extrinsic parameters (inliers). A pair is considered to agree with the extrinsic parameters if the reprojected error of that pair is smaller than the threshold $\epsilon_{error}$:  **Step 3.** Repeat Steps 1 and 2 until the number of inliers reaches a certain threshold or the number of repetitions is greater than *M*.  **Step 4.** Choose the extrinsic parameters that has the highest number of the inliers (the most stable) based on the method that we presented in Section 3.2.

**Algorithm 2:** Determine the list of key pair locations

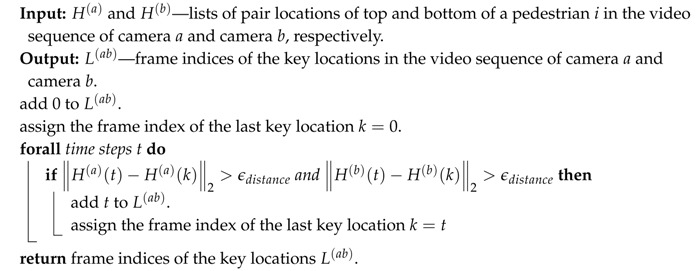



### 3.4. Automatically Estimates Extrinsic Parameters Based on Multiple Pedestrians

In [14], Guan et al. had to manually annotate the correspondences in the scene to calibrate the camera network. However, it is not really convenient for the customers to annotate the data, especially, if the number of cameras in the network is huge. Thus, we propose a simple and fast method to find the correspondences between different cameras. We use Openpose [16] to estimate 2D skeleton models of humans in the images. In practice, the estimated locations of the necks and feet (or hip) joints thus obtained are sometimes inconsistent between views (e.g., a different physical point is indicated the feet joints or hip joints in two views). The proposed calibration method is insensitive to this problem, as long as the number of observed skeletons is large enough. Otherwise, the results will be poor if people are observed in an insufficient number of locations (e.g., the method will fail if only a single person, always in the same position, is observed).

In controlled environments, these conditions can be easily enforced by providing instructions to the walking people. However, in uncontrolled environments with multiple pedestrians, we cannot order the people to walk by our instruction. In addition, people tend to pass the scene in a short amount of time such as walking along a straight line (insufficient number of locations). Hence, it is difficult to gather data in different locations of the scene for precise calibration.

To handle this problem, we propose an easy and robust brute-force method to solve the association problem (which pedestrian in one camera corresponds to an observation in another camera). First, we apply a simple object matching algorithm based on feature matching to track the pedestrians for each camera. Let $H_{i}^{\left(k\right)}=\left\{\left({\tilde{u}}_{\mathrm{bottom}}^{\left(k\right)}\left(m\right),{\tilde{u}}_{\mathrm{top}}^{\left(k\right)}\left(m\right)\right)\ldots ,\left({\tilde{u}}_{\mathrm{bottom}}^{\left(k\right)}\left(n\right),{\tilde{u}}_{\mathrm{top}}^{\left(k\right)}\left(n\right)\right)\right\}$ be the set of all locations of the top and bottom (feet or hip) of person *i* from frame *m* to frame *n* in camera *k* with k∈{a,b}.

Furthermore, let $H^{\left(k\right)}=\left\{H_{0}^{\left(k\right)},H_{1}^{\left(k\right)},\ldots ,H_{q}^{\left(k\right)}\right\}$ be a set of locations of the top and bottom of all pedestrians in the scene of camera *k* with *q* is the number of pedestrians in this scene. We compute all possible correspondences $C^{\left(ab\right)}$ between camera *a* and camera *b* by generating all pairs of elements from $H^{\left(a\right)}$ and $H^{\left(b\right)}$. Then, we calibrate the pair of cameras with each generated correspondences to estimate the matching rate as presented in the Algorithm 3. The matching rate represents the percentage of good reprojected results of the top and bottom positions of pedestrians where a good reprojected result means having the relative reprojection error less than threshold (equals 0.05 in our experiments).

**Algorithm 3:** Compute matching rate of the extrinsic parameters between camera *a* and camera *b*.

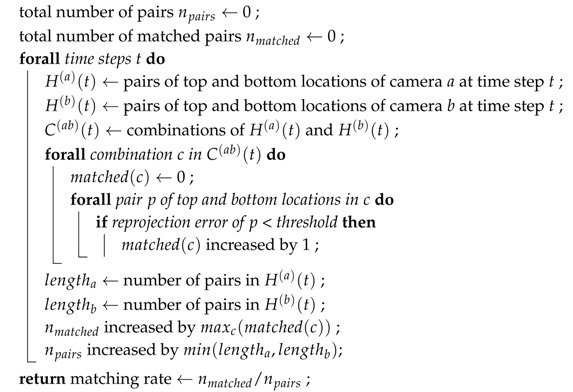



Finally, the top highest matching rate correspondences are selected to calibrate the pair of cameras. Thus, the difficulty of gathering data in uncontrolled environments also be solved by combining the sample locations of the highest matching rate correspondences from different spots of the scene. To avoid a combinatorial increase in the number of computations, the frames with too many pedestrians are removed. Moreover, the frames with too many pedestrians also have much worse pose estimation results as well as poor human tracking results. This makes it is not useful already to use these frames. Also, using the frames that have a very high number of people does not improve the accuracy of the calibration. Therefore, in practice, we can calibrate the camera network from the parts of video sequences which has a low enough number of pedestrians. In our experiments, we choose 5 pedestrians as the threshold, therefore the number of combination of correspondences for each frame is always less than 5! which does not take too much time to verify all possibilities. The brute-force approach can definitely be replaced by a random sampling scheme to make it runs faster. However, it may skip many appropriate correspondences for the calibration. Therefore, we decide to keep the brute-force approach to maintain the accuracy of the proposed method which is much more important for many applications in practice.

### 3.5. Extrinsic Calibration When Feet and Hip Joints Are Not Available

In Section 3.2 we proposed to estimate the normal vector using 2D image positions of the top and bottom pedestrians. As the feet and hip positions are not known, we propose to use line positions to do the estimation instead. Suppose a person moves to *N* different positions while keeping a fixed posture. Suppose that all cameras see the top of the person. At each time *t*, we first calculate ${\tilde{u}}_{\mathrm{top}}^{\left(k\right)}\left(t\right)$ and the centerline using the technique described above for camera *k*. We denote the projection of the centerline in the image as

(5) $$y=a^{k}\left(t\right)x+b^{k}\left(t\right).$$

Select two different points on that line and denote the normalized homogeneous image coordinates as ${\left(x_{1}^{\left(k\right)},ax_{1}^{\left(k\right)},1\right)}^{T}$ and ${\left(x_{2}^{\left(k\right)},ax_{2}^{\left(k\right)},1\right)}^{T}$. The cross product of these two vectors is ${\left(a^{k}\left(t\right)\left(x_{1}^{\left(k\right)}-x_{2}^{\left(k\right)}\right),x_{2}^{\left(k\right)}-x_{1}^{\left(k\right)},b^{k}\left(t\right)\left(x_{1}^{\left(k\right)}-x_{2}^{\left(k\right)}\right)\right)}^{T}$. By canceling out $\left(x_{1}^{\left(k\right)}-x_{2}^{\left(k\right)}\right)$, we define $m^{\left(k\right)}\left(t\right)={\left(-a^{\left(k\right)}\left(t\right),1,-b^{\left(k)(t\right)}\right)}^{T}$, which is the normal of the plane spanned by the camera center and the center line of the pedestrian. It is obvious that $e_{z}^{\left(k\right)}$ is on that plane, so that $m^{\left(k\right)}\left(t\right)$ and $e_{z}^{\left(k\right)}$ are orthogonal as Equation (4).

Therefore, $e_{z}^{\left(k\right)}$ can be determined in the same fashion as presented in Section 3.2 by using SVD decomposition of ${\left(m^{\left(k\right)}\left(t\right)\right)}^{T}$: $e_{z}^{\left(k\right)}$ is the singular vector which is corresponding to the lowest singular value. $e_{z}^{\left(k\right)}$ is also the normal of the plane composed by all the top positions when the person walks upright on a flat horizontal surface. Since all top positions of a single pedestrian lie on a plane when the person walks on a horizontal surface, there is the homography between any two views of the plane. Various methods [28,29,34] have been proposed to estimate the homography between two views given at least 4 non-collinear corresponding points and solve the structures from motion problem by decomposing the homography matrix. Weng et al. [28] produces two solutions of the extrinsic parameters by decomposing the homography matrix. However, the unique and proper solution cannot be obtained without additional knowledge of the scene. Here we propose a method to obtain the unique extrinsic parameters by decomposing the homography matrix with the estimated $e_{z}^{\left(k\right)}$.

**Pairwise Planar Homography:** as we select the coordinate frame of camera 1 as the world coordinate frame, we have

(6) $$r_{\mathrm{top}}^{\left(k\right)}\left(t\right)=R^{\left(k\right)}r_{\mathrm{top}}^{\left(1\right)}\left(t\right)+c^{\left(k\right)}$$

In the coordinate system of camera 1, the top of the pedestrian lies on the plane

(7) $${\left(n^{\left(1\right)}\right)}^{T}r_{\mathrm{top}}^{\left(1\right)}\left(t\right)=d^{\left(1\right)}\Leftrightarrow \frac{1}{d^{\left(1\right)}}{\left(n^{\left(1\right)}\right)}^{T}r_{\mathrm{top}}^{\left(1\right)}\left(t\right)=1,$$

with $d^{\left(1\right)}$ is the Euclidean distance between the center of camera 1 and the plane, and $n^{\left(1\right)}$ is the normal vector of the plane w.r.t the camera coordinate system of camera $C_{1}$. Substituting Equation (7) in Equation (6) gives

(8)rtop(k)t=R(k)rtop(1)t+c(k)1d(1)n(1)Trtop(1)t(9)=R(k)+c(k)1d(1)n(1)Trtop(1)t.

Since $r_{\mathrm{top}}^{\left(k\right)}\left(t\right)=Z_{\mathrm{top}}^{\left(k\right)}\left(t\right){\tilde{x}}_{\mathrm{top}}^{\left(k\right)}\left(t\right)$, and $r_{\mathrm{top}}^{\left(1\right)}\left(t\right)=Z_{\mathrm{top}}^{\left(1\right)}\left(t\right){\tilde{x}}_{\mathrm{top}}^{\left(1\right)}\left(t\right)$, Equation (9) leads to

(10) $${\tilde{x}}_{\mathrm{top}}^{\left(k\right)}\left(t\right)∼H^{\left(k\right)}{\tilde{x}}_{\mathrm{top}}^{\left(1\right)}\left(t\right),$$

where

(11) $$H^{\left(k\right)}=\left(R^{\left(k\right)}+c^{\left(k\right)}\frac{1}{d^{\left(1\right)}}{\left(n^{\left(1\right)}\right)}^{T}\right),$$

which is the homography matrix. Since the vectors from both sides of Equation (11) are parallel, their cross product is zero, which leads to

(12) $${\tilde{x}}_{\mathrm{top}}^{\left(k\right)}\left(t\right)\times \left(H^{\left(k\right)}{\tilde{x}}_{\mathrm{top}}^{\left(1\right)}\left(t\right)\right)=0,$$

with × representing the cross product.

Notice that $H^{\left(k\right)}$ depends only upon eight independent coefficients, i.e., the three rotation coefficients, the three coordinates of the translation, and the two parameters representing the orientation of the plane. Each point imposes two independent constraints on $H^{\left(k\right)}$, so we need at least four corresponding points (top positions) to solve uniquely for $H^{\left(k\right)}$. The four points should be in a general configuration in the plane (no three of them are collinear). Notice that $H^{\left(k\right)}$ can be recovered up to a scale factor, so we get the homography matrix in the form of

(13) $$H_{e}^{\left(k\right)}=\lambda \left(R^{\left(k\right)}+c^{\left(k\right)}\frac{1}{d^{\left(1\right)}}{\left(n^{\left(1\right)}\right)}^{T}\right).$$

**Decomposition of the Homography Matrix**: Once $H_{e}^{\left(k\right)}$ is obtained, we now discuss how to get extrinsic parameters by decomposing the homography matrix. It has been proven in the literature [28,29] that decomposing the homography matrix will give two candidate solutions for the extrinsic parameters. We will give detail about how to get the unique extrinsic parameters by using additional information.

Ma et al. [35] gave four solutions of the extrinsic parameters by SVD of the homography matrix, only two of which satisfy the positive depth constraint (the *Z* coordinate of the normal vector of the plane need to be positive, since the camera can see only points that are in front of it). Decomposition of the homography matrix proceeds as follows:

1. Normalization of the homography matrix. The normalized homography matrix is computed as
  (14) $$H^{\left(k\right)}=H_{e}^{\left(k\right)}/\sigma_{2}\left(H_{e}^{\left(k\right)}\right),$$
  where $\sigma_{2}\left(H_{e}^{\left(k\right)}\right)$ is the second largest singular value of $H_{e}^{\left(k\right)}$.
2. Compute the SVD of ${\left(H^{\left(k\right)}\right)}^{T}H^{\left(k\right)}$ as
  (15) $${\left(H^{\left(k\right)}\right)}^{T}H^{\left(k\right)}={\left(V^{\left(k\right)}\right)}^{T}S^{\left(k\right)}V^{\left(k\right)}.$$
  Define $v_{1}^{\left(k\right)}$, $v_{2}^{\left(k\right)}$, $v_{3}^{\left(k\right)}$ as the three column vectors of $V^{\left(k\right)}$, and $\sigma_{1}^{\left(k\right)}$, $\sigma_{2}^{\left(k\right)}$, $\sigma_{3}^{\left(k\right)}$ as the eigenvalues of $H^{\left(k\right)}$. Compute two more unit vectors by
  (16) $$u_{1}^{\left(k\right)}=\frac{\sqrt{1-{\left(\sigma_{3}^{\left(k\right)}\right)}^{2}}v_{1}^{\left(k\right)}+\sqrt{{\left(\sigma_{1}^{\left(k\right)}\right)}^{2}-1}v_{3}^{\left(k\right)}}{\sqrt{{\left(\sigma_{1}^{\left(k\right)}\right)}^{2}-{\left(\sigma_{3}^{\left(k\right)}\right)}^{2}}}$$
  and
  (17) $$u_{2}^{\left(k\right)}=\frac{\sqrt{1-{\left(\sigma_{3}^{\left(k\right)}\right)}^{2}}v_{1}^{\left(k\right)}-\sqrt{{\left(\sigma_{1}^{\left(k\right)}\right)}^{2}-1}v_{3}^{\left(k\right)}}{\sqrt{{\left(\sigma_{1}^{\left(k\right)}\right)}^{2}-{\left(\sigma_{3}^{\left(k\right)}\right)}^{2}}}.$$
3. Define matrices
  (18) $$\left.\begin{array}{cc} U_{1}^{\left(k\right)} & =\left[v_{2}^{\left(k\right)},u_{1}^{\left(k\right)},v_{2}^{\left(k\right)}\times u_{1}^{\left(k\right)}\right],\\ U_{2}^{\left(k\right)} & =\left[v_{2}^{\left(k\right)},u_{2}^{\left(k\right)},v_{2}^{\left(k\right)}\times u_{2}^{\left(k\right)}\right],\\ W_{1}^{\left(k\right)} & =\left[Hv_{2}^{\left(k\right)},Hu_{1}^{\left(k\right)},\left(Hv_{2}^{\left(k\right)}\right)\times \left(Hu_{1}^{\left(k\right)}\right)\right],\\ W_{2}^{\left(k\right)} & =\left[Hv_{2}^{\left(k\right)},Hu_{2}^{\left(k\right)},\left(Hv_{2}^{\left(k\right)}\right)\times \left(Hu_{2}^{\left(k\right)}\right)\right]. \end{array}\right.$$
  Finally the four possible solutions of extrinsic parameters and normal vector of the plane are calculated as
  (19) $$\begin{array}{ccc} R_{1}^{\left(k\right)} & = & W_{1}^{\left(k\right)}{\left(U_{1}^{\left(k\right)}\right)}^{T},\\ n_{1}^{\left(1\right)} & = & v_{2}^{\left(k\right)}\times u_{1}^{\left(k\right)},\\ c_{1}^{\left(k\right)}\frac{1}{d^{\left(1\right)}} & = & \left(H-R_{1}^{\left(k\right)}\right)n_{1}^{\left(1\right)}, \end{array}$$
  (20) $$\begin{array}{ccc} R_{2}^{\left(k\right)} & = & W_{2}^{\left(k\right)}{\left(U_{2}^{\left(k\right)}\right)}^{T},\\ n_{2}^{\left(1\right)} & = & v_{2}^{\left(k\right)}\times u_{2}^{\left(k\right)},\\ c_{2}^{\left(k\right)}\frac{1}{d^{\left(1\right)}} & = & \left(H-R_{2}^{\left(k\right)}\right)n_{2}^{\left(1\right)}, \end{array}$$
  (21) $$\begin{array}{ccc} R_{3}^{\left(k\right)} & = & R_{1}^{\left(k\right)},\\ n_{3}^{\left(1\right)} & = & -n_{1}^{\left(1\right)},\\ c_{3}^{\left(k\right)}\frac{1}{d^{\left(1\right)}} & = & -c_{1}^{\left(k\right)}\frac{1}{d^{\left(1\right)}}, \end{array}$$
  (22) $$\begin{array}{ccc} R_{4}^{\left(k\right)} & = & R_{2}^{\left(k\right)},\\ n_{4}^{\left(1\right)} & = & -n_{2}^{\left(1\right)},\\ c_{4}^{\left(k\right)}\frac{1}{d^{\left(1\right)}} & = & -c_{2}^{\left(k\right)}\frac{1}{d^{\left(1\right)}}. \end{array}$$

Notice that only two solutions of the above four solutions satisfy the positive depth constraint (i.e., the third coordinate of the normal vector for the plane should be positive since the camera can only see points that are in front of it). So either Equation (19) or Equation (21) is the possible solution. Similarly, either Equation (20) or Equation (22) is the possible solution. Once two possible solutions are obtained, we obtain the correct one as follows. From Equation (4), we estimate $e_{z}^{\left(1\right)}$, which is the normal of the plane composed by all the top positions of the pedestrian. $e_{z}^{\left(1\right)}$ should be equal to the estimated $n^{\left(1\right)}$ if there is no noise in the data. In practice, it rarely happens due to all kinds of noise. Thus we propose to use the angle between $e_{z}^{\left(1\right)}$ and $n^{\left(1\right)}$ as a criterion to get the correct $n^{\left(1\right)}$ and the corresponding rotation and translation parameters. The correct solution should have a smaller angle to $e_{z}^{\left(1\right)}$. Finally, to calibrate the camera network with the torsors of multiple pedestrians, we choose the highest matching rate correspondence for the calibration. In this case, we use the detected bounding boxes of the pedestrians which are extracted by YOLO [31] to determine the top and bottom of the pedestrians. We choose the center of the top edge and the center bottom edge of the bounding boxes as top and bottom of the pedestrians.

### 3.6. Joint Extrinsic Refinement for All Cameras in the Network

In Section 3.2, Section 3.3 and Section 3.4, we present the extrinsic calibration for the camera network in pairwise fashion. However, the extrinsic parameters of the cameras are obtained based on algebraic distance minimization without taking the property of the projective geometry of the cameras into account. Thus, it increases the triangulation error and projection error when we combine the information from all available cameras. To solve this problem, we jointly refine the extrinsic parameters of all cameras by minimizes the total reprojection error based on the method proposed in [14,36]). The objective function defines by the mean-squared discrepancy between the observed image positions of bottom and top of the pedestrian, and their reprojections. Finally, we optimize the extrinsic parameters by an iterative gradient descent procedure.

## 4. Experimental Results

### 4.1. Performance Measures

In order to evaluate the performance of our method with ground truth points, we compute the triangulation error ($\delta r^{\left(w\right)}$), projection error ($\delta u^{\left(p\right)}$), and reprojection error ($\delta u^{\left(r\right)}$) [14]. In practice, the ground truth points are not always available to measure the performance of the calibration. Thus, we can only measure the calibration by computing reprojection error based on the top (or the bottom) positions of detected pedestrians. However, different cameras have different resolutions. Moreover, the height of the pedestrians at different locations in an image is different. Let *N* be the number of ground truth 3D sample points and *K* is the number of cameras in the network. We define the relative reprojection error as follows:

(23) $$\delta u^{\left(rr\right)}=\frac{1}{MK}\sum_{m=1}^{M}\sum_{k=1}^{K}\frac{{∥u_{mk}-{\hat{u}}_{mk}^{\left(rr\right)}∥}_{2}}{h_{mk}},$$

where *N* be the number of ground truth 3D sample points, *K* is the number of cameras in the network, *M* is the number of pedestrians, $h_{m}$ is the height in the image of person *m*-th in the camera *k*-th. $u_{mk}$ is the observed pixel coordinates of the top (or the bottom) of person *m*-th in the camera *k*-th. ${\hat{u}}_{mk}^{\left(rr\right)}$ is the estimated location of the top (or the bottom), which are obtained through reprojection.

### 4.2. Calibration with Controlled Environment

**Figure 3:**
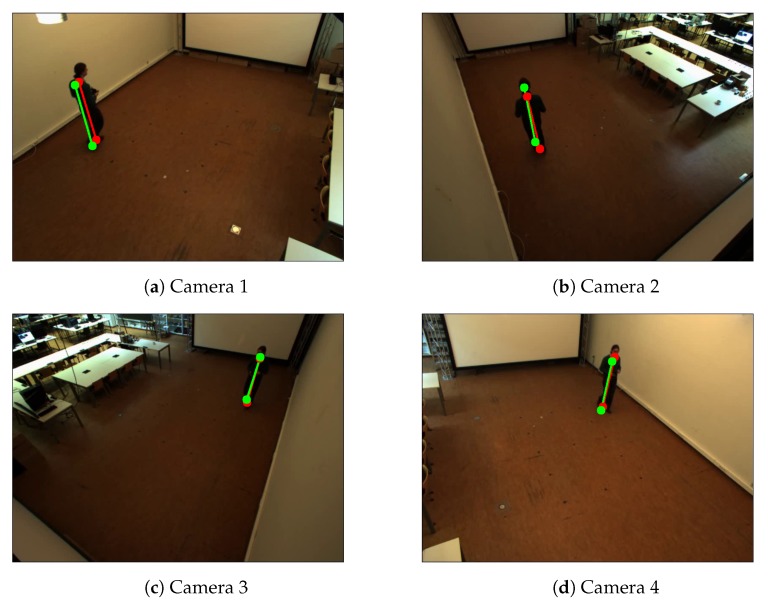
Example of detected the bottom and top positions of the pedestrians of Camera Network 1—single person walking in an empty room. Red color represents detected positions by the human pose estimation method. Green color represents the detected reprojected positions.

**Calibration with single person.** We evaluate our method with a multi-camera tracking system composed of four side-view cameras. For simplicity, we call it Camera Network 1. The cameras were mounted at a height of about 3 m at each corner of a room (8.6 m by 4.8 m). The resolutions of the all videos are 780 by 580 pixel (Figure 3). We obtain the intrinsic parameters by [7]. We compare our method to the calibration method of Hödlmoser et al. [24] and Guan et al. [14].

Figure 3 shows an example of the detected bottom and top positions of the pedestrians of the person in a scene. For single person case, we apply the refinement method which proposed in [14] to obtain the final extrinsic parameters (Table 2). Table 2 shows that our method has slightly more accurate results than state-of-the-art methods. The differences in accuracy among different methods are small because this case is the simplest situation. Therefore, most of the existing methods can achieve very high accuracy on these video sequences.

In addition, the proposed method also requires a very short amount of time to collect the sample data for the calibration. As shown in Table 3, to collect enough data for the calibration, the person only needs to walk around the room with the total accumulated moving distance is around 20 m (by normal walking speed). This is the distance of walking around the room 2–3 times. Thus, the proposed method is fast and convenient for users to calibrate camera networks.

**Figure 4:**
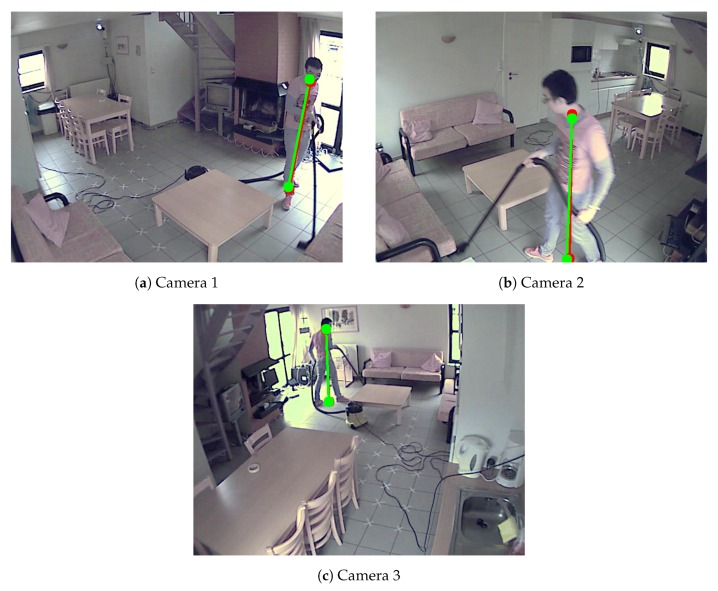
Example of detected the bottom and top positions of the pedestrians of Camera Network 2—single person doing household work in an kitchen room. Red color represents detected positions by the human pose estimation method. Green color represents the detected reprojected positions.

**Calibration based on a single person in a mildly occluded scene.** In order to show that our method works in a complex real-life environment room setup, we also evaluate our method on a setup with three cameras in a kitchen (Figure 4). The cameras were mounted at a height of about 2 m at different corners of a room. The resolutions of the videos are 640 by 480 pixels. We call it Camera Network 2 for simplicity. Table 2 shows that our method outperforms the method proposed by Guan et al. [14]. Note that we also recorded a scene where the person was cleaning the kitchen floor. Despite the movement and occlusions while cleaning the floor, the proposed method still produces a reasonable accuracy (the triangulation error is less than 10 cm) as shown in Table 4 and Table 5. It shows the stability and robustness of our method to the occlusion.

**Robust extrinsic calibration.** In Table 2, we present the calibration results based on different locations in the scene to evaluate the performance of the proposed method in a general sense. However, in practice, we have to collect the data consecutively rather than select arbitrary locations in space.

Thus, we also conduct another experiment of camera calibration based on a segment with regards to the amount of time that we use to collect data. Table 6 shows the successful rate to have accurate extrinsic parameters with the proposed method with all samples and a random-sample scheme. We apply the refinement method which proposed in [14] to obtain the final extrinsic parameters in Table 6. While cleaning the floor, we cannot keep one posture from the beginning until the end. Typically, we have to lean forward or to kneel for cleaning different places in the room. Thus, the result of our method by using all available locations on CN2—cleaning the floor sequences (Table 4) are degraded dramatically. By applying the random sample scheme, the proposed method can eliminate the locations produced from improper postures. Therefore, this improves both the successful percentage and the accuracy of the calibration. Table 1, Table 5 and Table 6 also show that the random sample scheme improves the successful percentage of the calibration and the accuracy of the calibration in the case that the pedestrian was walking with the same posture.

**Figure 5:**
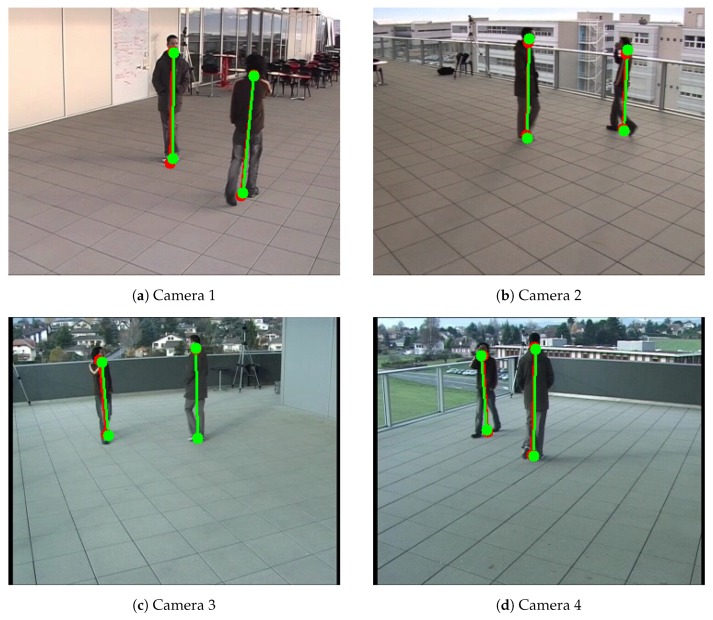
Example of detected the bottom and top positions of the pedestrians of Camera Network 3—EPFL-Terrace dataset [37]. Red color represents detected positions by the human pose estimation method. Green color represents the detected reprojected positions.

**Calibration with multiple walking people.** We evaluate our calibration method on the EPFL-Terrace sequences [37], which is a public multi-camera pedestrian video dataset (Figure 5). This dataset includes two sequences and 7 subjects, which were shot outside our building on a terrace with four DV cameras. In this paper, we call it Camera Network 3 for simplicity.

It only takes approximately 270 s and 210 s on EPFL-Terrace dataset and CN3 to solve the correspondences and obtain the extrinsic parameters, respectively (we implemented the code to run on Intel(R) Core(TM) i7-8086K CPU @ 4.00GHz with Python 3). Table 7 also shows very high accuracy results for very challenging multiple people sequences. It proves the propsed scheme to correspondences in the video sequences can deal with outliers and failed tracking results to provide very appropriate pairs of correspondences.

**Figure 6:**
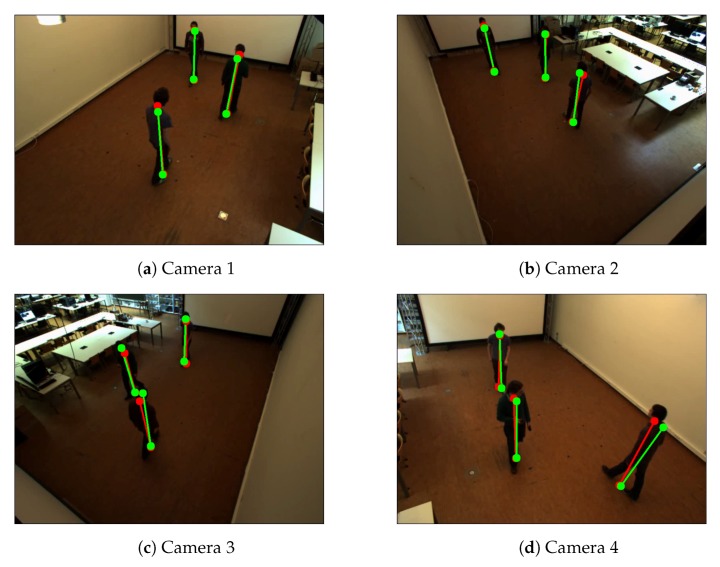
Example of detected the bottom and top positions of the pedestrians of Camera Network 1—multiple people walking in an empty room. Red color represents detected positions by the human pose estimation method. Green color represents the detected reprojected positions.

**Figure 7:**
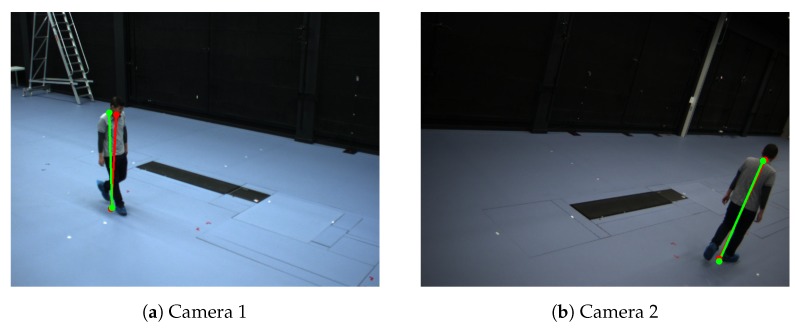

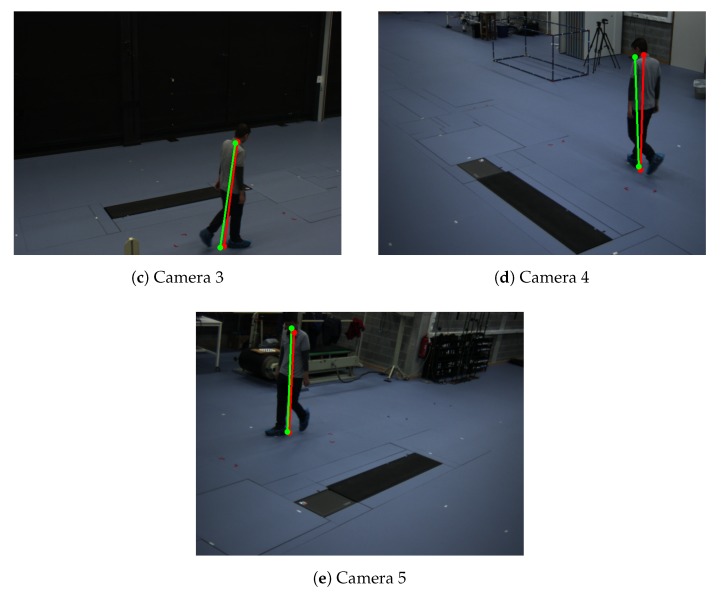
Example of detected the bottom and top positions of the pedestrians of Camera Network 4—single person in an empty room. Red color represents detected positions by the human pose estimation method. Green color represents the detected reprojected positions.

**Figure 8:**
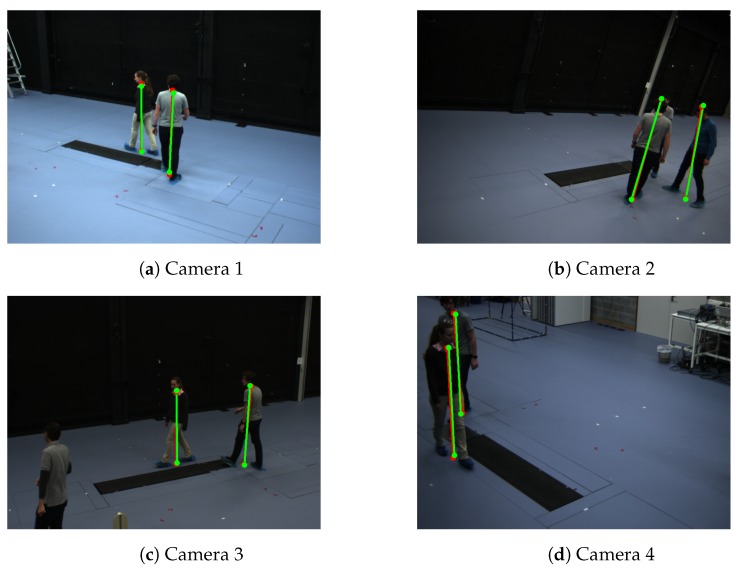

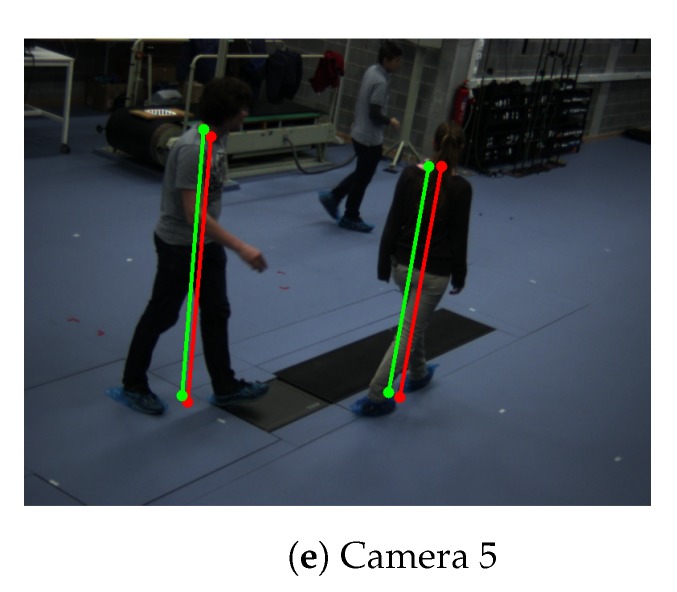
Example of detected the bottom and top positions of the pedestrians of Camera Network 4—single person in an empty room. Red color represents detected positions by the human pose estimation method. Green color represents the detected reprojected positions.

**Comparison between calibration based on single walking person and calibration based on multiple walking.** We evaluate the proposed camera calibration based on multiple pedestrians by calibrating the Camera Network 1 with an extra sequence to compare with the result of the calibration based on a single person. This sequence was shot with 3 subjects walking at the same time in the empty room (Figure 6). We calibrated Camera Network 4—CN4 (Figure 7 and Figure 8) to compare the performance of the calibration in both single person and multiple people situations. This network has five cameras, three of which are mounted at a height of about 3 m, and two cameras were mounted at a height of around 2 m. The resolution of all cameras is 780 by 580 pixels. In the experiments, we also change the orientation and locations of the cameras in the Camera Network 4 to verify the performance of the proposed method from different viewpoints.

Table 2 and Table 8 show the results of the proposed method in the case that there is only one subject in the scene of Camera Network 1 and Camera Network 4, respectively. Table 9 shows the results of the proposed method in multiple walking people case which is reasonably close to the calibration results in the single person case. It shows that the proposed method works well in both cases.

In general, using the feet to calibrate the camera network produce much better results in single-person cases because the $he_{z}^{\left(k\right)}$ vector is much longer which makes centerline vector estimation becomes more consistent. In the single person case, we can collect the data of the walking person continuously. Even in the case of occlusion, we can easily associate the data because they belong to only one person. However, in multiple pedestrian case, the data of one person normally are scattered and labeled with different indices due to the occlusion and tracking failures. Thus, the number of useful samples that we could extract in the same amount of time can be reduced. However, the proposed extrinsic calibration method based on multiple walking people in Section 3.4 still obtains the accuracy which is close to the single person case (Table 8 and Table 9). Finally, using hip joints also improves the calibration results where the bottom parts of the pedestrians are hard to observe (e.g., S1 and S5 in Table 9).

### 4.3. Calibration with Uncontrolled Environment

**Figure 9:**
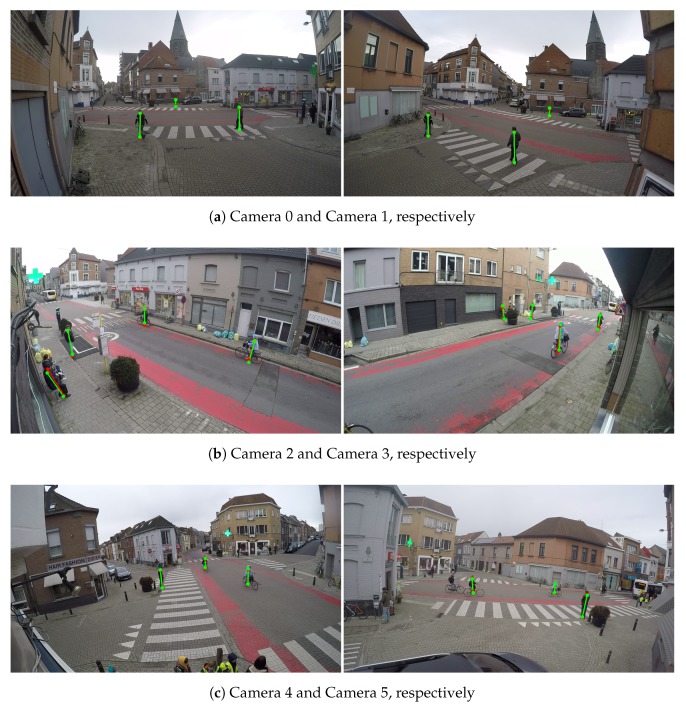
Example of detected the bottom and top positions of the pedestrians of Camera network 5—multiple pedestrians at an intersection. Red color represents detected positions by the human pose estimation method. Green color represents the detected reprojected positions.

**Calibration at intersections.** To show that our method can be applied to a real-life situation, we also recorded several video sequences at an intersection in Ghent to evaluate the proposed method (Figure 9). The pedestrians in this scene are quite small (about 60 pixels height). We call it Camera Network 5 for simplicity.

Table 2 shows our method has reasonably low error among different circumstances. However, in the intersection case, the pedestrians appear in some regions that are too small to detect by the human pose estimation. In addition, when the trajectories of the pedestrians are too short, the estimated extrinsic parameters normally have high relative reprojection error. Thus, the matching rates of them are too small to be selected by the proposed method in Section 3.4. Hence, the proposed method also could not find the samples at some regions of the scene, which lead which leads to a higher relative reprojection error for those regions. However, the errors in these regions are still small enough for applications like multi-camera tracking for the intersection scenes.

### 4.4. Calibration with Crowded Scene

**Figure 10:**
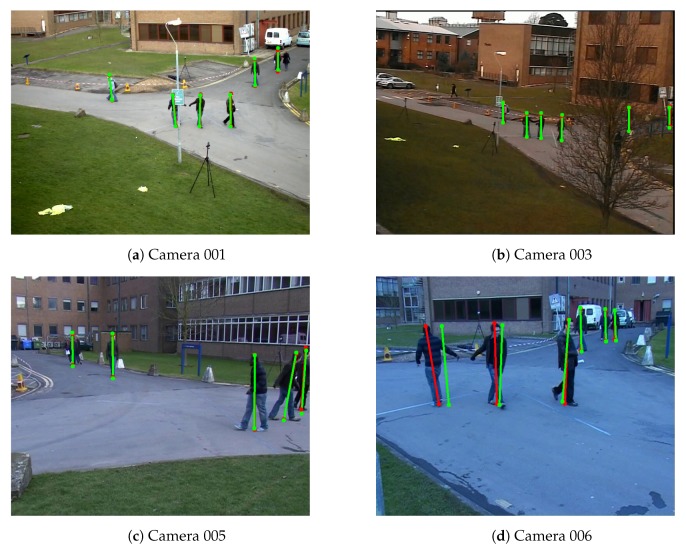

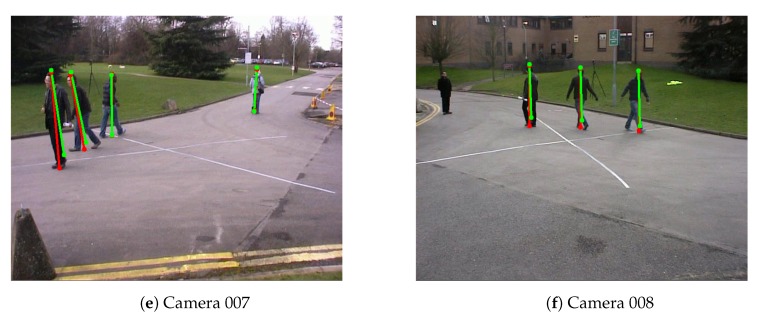
Example of detected the bottom and top positions of the pedestrians of Camera Network 6—PETS2009-S2L1 dataset. Red color represents detected positions by the human pose estimation method. Green color represents the detected reprojected positions.

In this work, we also apply the proposed method on PETS2009-S2L1 dataset (CN6), which is a crowded scene with multiple pedestrians [38]. Due to the small resolution of the pedestrians in the scenes, the results of OpenPose [15,16] are not stable enough to calibrate the camera network. Thus, we only use the detected bounding boxes of the pedestrians which are extracted by YOLO [31]. We choose the center of the top edge and the center bottom edge of the bounding boxes as top and bottom of the pedestrians. We select the coordinate system of the camera 001 as the world coordinate system. To make the method work on this dataset, we also remove the limitation of the number of pedestrians that appear in the scene. Because of the orientation of the camera view 004, the tracking algorithm has a poor performance on this scene. Note that, the camera view 002 of the dataset is not available. Therefore, the result of the proposed method is not available for this scene. Even though the proposed method only works best for the scene that does not have so many pedestrians, the relative reprojection errors showed in Table 10 are still reasonably low. Figure 10 shows the example of the detected top and bottom of PETS2009-S2L1 dataset.

### 4.5. Calibration with without Feet and Hip Joints

**Figure 11:**
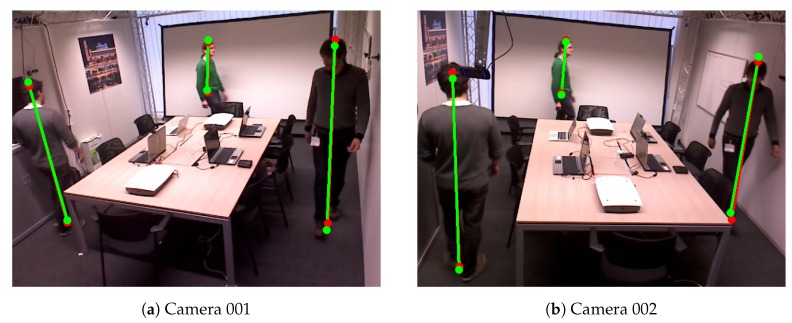

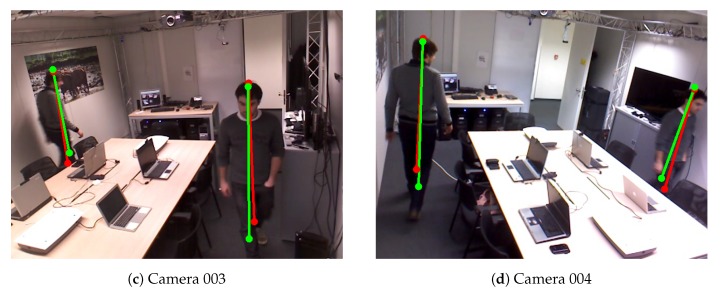
Example of detected the bottom and top positions of the pedestrians of Camera Network 7. Red color represents detected positions by the human pose estimation method. Green color represents the detected reprojected positions.

We evaluated the propsed method in Section 3.5 with Camera Network 7—a heavily occluded scene in an office (Figure 11). Since the scene is heavily occluded, the feet information is not enough for the extrinsic calibration based on feet joints. The extrinsic calibration based on hip joints is also not always available for all camera views. Table 11 shows that the proposed extrinsic calibration based on just the position of the top and the centerline can still obtain the accuracy that is very close to the extrinsic calibration based on hip joints. In the case of camera pair 003-004, the extrinsic calibration based on hip joints cannot collect enough data to estimate extrinsic parameters on single person sequences. On the other hand, the proposed method in Section 3.5 is still able to produce the extrinsic parameters with low relative reprojection errors. Figure 11 shows the example of the detected top and bottom of Camera Network 7.

## 5. Conclusions

In this paper, we present a simple and robust method to leverage the human pose estimation for the computation of 3D positions of the top and bottom of the pedestrians. To handle the case where multiple pedestrians are in the scene, we also developed a brute-force method to select appropriate top and bottom locations for the extrinsic camera calibration. For indoor camera networks which are intended for people surveillance, the feet of pedestrians are usually occluded by the furniture. This is the degenerate for most of the current state-of-the-art calibration methods due to the coplanarity of all the positions of the top of a single pedestrian. To the best of our knowledge, no work exists to deal with camera network calibration for this specific scenario. We proposed the extrinsic calibration by using a walking human as the calibration object, assuming only the top of the pedestrian and centerline of the person are visible. Thus, the proposed method can be very useful for many of the existing indoor multi-camera visual surveillance systems. The proposed method can work well in various environments as well as robust against occlusion compared to state-of-the-art methods. More importantly, the proposed method can work completely automatically without manually selecting proper input data for the calibration method. In the future, we will investigate a regional selection method to handle the case where the walking trajectory is too short.

## Figures and Tables

**Table 1:** The calibration results on Camera Network 1 (CN1) within 1000 experiments of the proposed method. $\delta r^{\left(w\right)}$, $\delta u^{\left(p\right)}$, $\delta u^{\left(r\right)}$, and $\delta u^{\left(rr\right)}$ denotes the triangulation error, projection error, reprojection error, and relative reprojection error, respectively.

Collecting Data Time (s)	10	20	30	40	50
Using all Locations					
**$\delta r^{\left(w\right)}$ (cm)**	5.203	2.443	1.655	1.540	1.533
**$\delta u^{\left(p\right)}$ (pixel)**	15.005	9.006	4.863	4.648	4.627
**$\delta u^{\left(r\right)}$ (pixel)**	52.804	7.544	4.581	4.341	4.316
Random Samples					
**$\delta r^{\left(w\right)}$ (cm)**	2.465	1.742	1.582	1.585	1.544
**$\delta u^{\left(p\right)}$ (pixel)**	5.952	4.839	4.670	4.613	4.595
**$\delta u^{\left(r\right)}$ (pixel)**	5.435	4.496	4.355	4.300	4.280

**Table 2:** Comparison between our method, the method of Guan et al. [14], and the method of Hödlmoser et al. [24] on Camera Network 1 (CN1), Camera Network 2 (CN2). We randomly select 20 locations of the pedestrians in the scene to calibrate the CN1, and CN2. $\delta r^{\left(w\right)}$, $\delta u^{\left(p\right)}$, $\delta u^{\left(r\right)}$, and $\delta u^{\left(rr\right)}$ denotes the triangulation error, projection error, reprojection error, and relative reprojection error, respectively. The bold numbers are the best results among the mentioned methods.

	Proposed Method (Feet)		Proposed Method (Hip)		Guan et al. [14]		Hödlmoser et al. [24]	
	**CN1**	**CN2**	**CN1**	**CN2**	**CN1**	**CN2**	**CN1**	**CN2**
$\delta r^{\left(w\right)}$ (cm)	1.33	**2.2**	1.45	**2.2**	**1.30**	3.16	**1.30**	63.7
$\delta u^{\left(p\right)}$ (pixel)	**3.98**	**5.8**	4.60	**5.8**	4.14	6.72	4.15	106.4
$\delta u^{\left(r\right)}$ (pixel)	**3.76**	**5.0**	4.33	**5.0**	4.09	6.20	4.09	104.3
$\delta u^{\left(rr\right)}$—top (%)	**1.8**	**1.7**	**1.8**	**1.7**	1.9	7.0	1.9	43
$\delta u^{\left(rr\right)}$—bottom (%)	**2.8**	**2.0**	**2.8**	**2.0**	3.0	4.1	3.0	39

**Table 3:** Success percentages (the triangulation error is below 15 cm) within 1000 experiments of the proposed method for the Camera Network 1.

**Accumulated Moving Distance (cm)**	650	1000	2000	2500	4000
**Successful Percentage**	0.35	0.83	0.97	0.99	1.0

**Table 4:** The calibration results on Camera Network 2 (CN2) within 1000 experiments of the proposed method. The table shows the results of our method on video sequence where the subject was cleaning the room. $\delta r^{\left(w\right)}$, $\delta u^{\left(p\right)}$, $\delta u^{\left(r\right)}$, and $\delta u^{\left(rr\right)}$ denotes the triangulation error, projection error, reprojection error, and relative reprojection error, respectively.

Collecting Data Time (s)	10	20	30	40	50
Using all Locations					
**$\delta r^{\left(w\right)}$ (cm)**	17.979	14.245	11.940	10.448	11.198
**$\delta u^{\left(p\right)}$ (pixel)**	214.329	182.073	364.136	115.872	165.385
**$\delta u^{\left(r\right)}$ (pixel)**	196.218	129.085	289.050	104.330	90.784
Random Samples					
**$\delta r^{\left(w\right)}$ (cm)**	5.320	4.511	3.041	3.243	2.912
**$\delta u^{\left(p\right)}$ (pixel)**	35.694	27.482	13.196	13.649	12.623
**$\delta u^{\left(r\right)}$ (pixel)**	37.039	36.265	12.655	12.592	12.099

**Table 5:** The calibration results on Camera Network 2 (CN2) within 1000 experiments of the proposed method. The table shows the results of our method on video sequence where the subject was walking in the room. $\delta r^{\left(w\right)}$, $\delta u^{\left(p\right)}$, $\delta u^{\left(r\right)}$, and $\delta u^{\left(rr\right)}$ denotes the triangulation error, projection error, reprojection error, and relative reprojection error, respectively.

Collecting Data Time (s)	10	20	30	40	50
Using All Locations					
**$\delta r^{\left(w\right)}$ (cm)**	4.499	3.602	2.689	2.504	2.456
**$\delta u^{\left(p\right)}$ (pixel)**	37.253	22.162	15.705	10.922	9.818
**$\delta u^{\left(r\right)}$ (pixel)**	39.925	21.332	13.780	10.096	8.927
Random Samples					
**$\delta r^{\left(w\right)}$ (cm)**	2.618	2.224	2.144	2.169	2.161
**$\delta u^{\left(p\right)}$ (pixel)**	14.634	11.331	9.198	7.933	7.156
**$\delta u^{\left(r\right)}$ (pixel)**	15.643	11.242	8.783	7.412	6.567

**Table 6:** Success percentages (the triangulation error is below 15 cm) within 1000 experiments of the proposed method which is conducted with the Camera Network 1 (CN1), and Camera Network 2 (CN2).

Collecting Data Time (s)	10	20	30	40	50
All locations					
**CN1—Successful Percentage**	0.896	0.972	0.995	1.0	1.0
**CN2 (cleaning the Floor)—Successful Percentage**	0.339	0.489	0.539	0.62	0.607
**CN2 (walking)—Successful Percentage**	0.836	0.929	0.980	1.0	1.0
Random Samples					
**CN1—Successful Percentage**	0.975	0.998	1.0	1.0	1.0
**CN2 (Cleaning the Floor)—Successful Percentage**	0.590	0.736	0.786	0.807	0.868
**CN2 (Walking)—Successful Percentage**	0.910	0.978	0.999	1.0	1.0

**Table 7:** The calibration results on EPFL-Terrace (CN3) and Camera Network 5 (CN5). For EPFL-Terrace (CN3) and Camera Network 5 (CN5), we apply the proposed method on the first half of the video to obtain the extrinsic parameters. $\delta r^{\left(w\right)}$, $\delta u^{\left(p\right)}$, $\delta u^{\left(r\right)}$ are not available for these sequences. Thus, the $\delta u^{\left(rr\right)}$ denotes relative reprojection error is the only one available for these sequences.

	Proposed Method		Proposed Method	
	(Feet)		(Hip)	
	**CN3**	**CN5**	**CN3**	**CN5**
$\delta u^{\left(rr\right)}$—top (%)	2.1	12.6	2.1	12.6
$\delta u^{\left(rr\right)}$—bottom (%)	2.3	17.2	2.1	12.6

**Table 8:** Calibration results of camera calibration based on single person of the Camera Network 4. $\delta r^{\left(w\right)}$, $\delta u^{\left(p\right)}$, $\delta u^{\left(r\right)}$, and $\delta u^{\left(rr\right)}$ denotes the triangulation error, projection error, reprojection error, and relative reprojection error, respectively.

	Proposed Method (Feet)					Proposed Method (Hip)				
	**S1**	**S2**	**S3**	**S4**	**S5**	**S1**	**S2**	**S3**	**S4**	**S5**
$\delta r^{\left(w\right)}$ (cm)	5.14	3.88	5.22	4.92	8.52	5.60	6.71	6.99	7.66	8.09
$\delta u^{\left(p\right)}$ (pixel)	6.04	5.52	6.31	6.21	10.67	6.35	8.39	8.92	9.84	10.86
$\delta u^{\left(r\right)}$ (pixel)	2.21	3.40	2.09	2.27	4.74	2.10	3.71	3.96	3.53	5.55
$\delta u^{\left(rr\right)}$—top (%)	2.3	2.2	2.4	2.7	3.1	2.8	2.2	2.6	2.7	2.7
$\delta u^{\left(rr\right)}$—bottom (%)	1.7	2.8	1.9	2.3	2.5	2.2	2.5	2.1	3.1	3.3

**Table 9:** Calibration results of camera calibration based on multiple walking people of the Camera Network 1 and the Camera Network 4. Note that, for Camera Network 1, we also apply the refinement method which proposed in [14] to obtain the final extrinsic parameters. $\delta r^{\left(w\right)}$, $\delta u^{\left(p\right)}$, $\delta u^{\left(r\right)}$, and $\delta u^{\left(rr\right)}$ denotes the triangulation error, projection error, reprojection error, and relative reprojection error, respectively.

	Proposed Method						Proposed Method					
	(Feet)						(Hip)					
	**CN1**	**S1**	**S2**	**S3**	**S4**	**S5**	**CN1**	**S1**	**S2**	**S3**	**S4**	**S5**
$\delta r^{\left(w\right)}$ (cm)	4.75	7.79	6.49	5.13	5.84	10.24	1.45	5.84	7.85	8.15	9.37	7.25
$\delta u^{\left(p\right)}$ (pixel)	5.23	10.52	7.53	6.22	6.55	11.58	4.61	7.09	9.20	10.03	10.66	8.71
$\delta u^{\left(r\right)}$ (pixel)	3.08	5.37	1.88	2.49	1.57	2.88	4.33	3.40	3.85	3.73	3.58	3.93
$\delta u^{\left(rr\right)}$—top (%)	2.1	5.8	2.2	2.9	2.7	6.4	2.5	3.5	2.8	4.4	3.4	3.3
$\delta u^{\left(rr\right)}$—bottom (%)	2.2	5.8	1.8	2.0	2.0	5.7	2.3	2.6	1.7	2.5	2.2	2.2

**Table 10:** Calibration results of camera calibration based on multiple walking people of the Camera Network 6. $\delta u^{\left(rr\right)}$ denotes the relative reprojection error.

	001-003	001-005	001-006	001-007	001-008
$\delta u^{\left(rr\right)}$—Top (%)	2.5	5.1	8.6	7.4	6.0
$\delta u^{\left(rr\right)}$—Bottom (%)	1.9	5.9	7.3	12.5	10.0

**Table 11:** Calibration results of camera calibration based on multiple walking people of the Camera Network 7. The extrinsic parameters was estimated based on the proposed method in Section 3.2 hip joints and the proposed method in Section 3.5 without both hip and feet information. $\delta u^{\left(rr\right)}$ denotes the relative reprojection error.

	Single Person				Multiple People			
	**001-002**		**003-004**		**001-002**		**003-004**	
	**Without Hip**	**Hip**	**Without Hip**	**Hip**	**Without Hip**	**Hip**	**Without Hip**	**Hip**
$\delta u^{\left(rr\right)}$—top (%)	2.8	1.2	7.7	N/A	2.7	1.4	1.3	1.2
$\delta u^{\left(rr\right)}$—bottom (%)	6.7	6.4	8.4	N/A	7.5	8.5	7.6	10.6

## Abbreviations

The following abbreviations are used in this manuscript:

RANSAC	Random sample consensus
2D	Two-dimensional
3D	Three-dimensional
SVD	Singular Value Decomposition
